# Head Down Tilt Bed Rest Plus Elevated CO_2_ as a Spaceflight Analog: Effects on Cognitive and Sensorimotor Performance

**DOI:** 10.3389/fnhum.2019.00355

**Published:** 2019-10-17

**Authors:** Jessica K. Lee, Yiri De Dios, Igor Kofman, Ajitkumar P. Mulavara, Jacob J. Bloomberg, Rachael D. Seidler

**Affiliations:** ^1^Department of Applied Physiology and Kinesiology, College of Health and Human Performance, University of Florida, Gainesville, FL, United States; ^2^German Aerospace Center, Cologne, Germany; ^3^KBRWyle, Houston, TX, United States; ^4^Johnson Space Center, NASA, Houston, TX, United States; ^5^Department of Neurology, University of Florida, Gainesville, FL, United States

**Keywords:** cognition, sensorimotor, CO_2_, bed rest, spaceflight, SANS (Spaceflight Associated Neuro-ocular Syndrome)

## Abstract

Long duration head down tilt bed rest (HDBR) has been widely used as a spaceflight analog environment to understand the effects of microgravity on human physiology and performance. Reports have indicated that crewmembers onboard the International Space Station (ISS) experience symptoms of elevated CO_2_ such as headaches at lower levels of CO_2_ than levels at which symptoms begin to appear on Earth. This suggests there may be combinatorial effects of elevated CO_2_ and the other physiological effects of microgravity including headward fluid shifts and body unloading. The purpose of the current study was to investigate these effects by evaluating the impact of 30 days of 6° HDBR and 0.5% CO_2_ (HDBR + CO_2_) on mission relevant cognitive and sensorimotor performance. We found a facilitation of processing speed and a decrement in functional mobility for subjects undergoing HDBR + CO_2_ relative to our previous study of HDBR in ambient air. In addition, nearly half of the participants in this study developed signs of Spaceflight Associated Neuro-ocular Syndrome (SANS), a constellation of ocular structural and functional changes seen in approximately one third of long duration astronauts. This allowed us the unique opportunity to compare the two subgroups. We found that participants who exhibited signs of SANS became more visually dependent and shifted their speed-accuracy tradeoff, such that they were slower but more accurate than those that did not incur ocular changes. These small subgroup findings suggest that SANS may have an impact on mission relevant performance inflight via sensory reweighting.

**New And Noteworthy:**

We examined the effects of long duration head down tilt bed rest coupled with elevated CO_2_ as a spaceflight analog environment on human cognitive and sensorimotor performance. We found enhancements in processing speed and declines in functional mobility. A subset of participants exhibited signs of Spaceflight Associated Neuro-ocular Syndrome (SANS), which affects approximately one in three astronauts. These individuals increased their visual reliance throughout the intervention in comparison to participants who did not show signs of SANS.

## Introduction

Successful spaceflight missions depend upon high levels of human performance. However, it is well established that astronauts experience altered sensory perception (Kornilova, 1997a; Clement et al., 2013) and declines in manual control, balance and locomotion both in- and post-flight (Lackner and DiZio, 1996; Kornilova, 1997b; Reschke et al., 1998; Bock et al., 2003, 2010). Moreover, astronauts have also reported “space fog” (Welch et al., 2009), including mental slowing, poor concentration, and slowed performance because of “feeling different than usual” (Kanas and Manzey, 2008).

Body unloading, altered vestibular signals, and fluid shifts toward the head from microgravity are thought to contribute to sensorimotor declines (Hargens and Richardson, 2009). In addition, crewmembers residing in the confined compartment of the International Space Station (ISS) are exposed to elevated ambient CO_2_ levels averaging 0.5%, which is more than ten times greater than terrestrial levels (0.04%) (Law et al., 2014). The environment aboard the ISS poses a potential risk to mission success and the long-term health of astronauts, as prolonged exposure to elevated ambient CO_2_ results in increased cerebral blood flow and mild performance impairments (Manzey and Lorenz, 1998; Satish et al., 2012; Fisk et al., 2013; Allen et al., 2015, 2018). Moreover, space station crewmembers have exhibited symptoms of elevated CO_2_ exposure such as headaches occurring at a more mild level of CO_2_ elevation than for headache symptoms in Earth-based studies of elevated CO_2_ (Law et al., 2014). Thus, there may be an interactive effect of elevated CO_2_ and the fluid shifts toward the head which we (Lee et al., 2019) and others (Roberts et al., 2017) have shown occur with spaceflight.

Long duration, head down tilt bed rest (HDBR) has been widely used to simulate the physiological impacts of microgravity such as arterial pressure changes, unloading of the lower body, and cephalad fluid shifts (Hargens and Vico, 2016). HDBR has been shown to result in decreased postural stability and functional mobility (Reschke et al., 2009; Mulder et al., 2014; Koppelmans et al., 2015, 2017; Miller et al., 2018; Mulavara et al., 2018). Moreover, a previous study has shown that prolonged exposure (26 days) to moderately elevated CO_2_ (0.7 and 1.2% concentration) negatively impacts visuomotor function (Manzey and Lorenz, 1998).

We administered the same cognitive and sensorimotor assessments here as we previously applied in an ambient air HDBR study (Koppelmans et al., 2015), allowing us to examine the combined effects of elevated HDBR + CO_2_ relative to HDBR alone. This comparison is somewhat exploratory, however, as the duration of HDBR differed between the two studies (experimental protocols in both cases were established by NASA’s Flight Analogs Program). Here, our primary aim was to investigate the effects of 6° HDBR with sustained exposure to elevated CO_2_ levels (0.5%, HDBR + CO_2_) on a range of cognitive and sensorimotor tests. Basner et al. (2017) recently reported altered cognitive response strategies (speed-accuracy trade-offs) as a result of acute exposure (i.e., 26.5 h) to 0.5% CO_2_ combined with 12° HDBR. In light of these findings, we hypothesized that subjects would show increased response speed and diminished accuracy on a series of cognitive assessments during 30 days of HDBR + CO_2_.

Approximately one third of astronauts who complete long-duration missions (Lee et al., 2016) manifest optic nerve and/or ocular changes, a condition referred to as Spaceflight Associated Neuro-ocular Syndrome (SANS) (Mader et al., 2011). Interestingly, at the end of the HDBR + CO_2_ intervention, five of the 11 participants in the current study showed bilateral optic disc edema, a sign of SANS, with Modified Frisèn Scale grades between one (*n* = 4) and two (*n* = 1) (Laurie et al., 2019). This was the first time that signs of SANS have been seen in HDBR; the finding provided us with a unique opportunity to further characterize the performance profiles of the participants who did and did not develop signs of SANS. The subgroup comparison between those who showed signs of SANS and did not (hereinafter referred to as SANS and NoSANS) will be carried out as a secondary aim.

## Materials and Methods

### Study Design and Participants

In the present analyses we compared two groups, one that underwent a 70 day, 6° HDBR study (HDBR group) and another that underwent a 30 day, 6° HDBR study coupled with an elevation of 0.5% CO_2_ (HDBR + CO_2_). In both groups, we administered a battery of cognitive and sensorimotor tests before, during, and after the bedrest period.

#### HDBR + CO_2_

Our experiment was implemented within the larger VaPER (Visual impairment intracranial pressure and Psychological:envihab Research) study which was conducted at:envihab, an environmental medicine research facility at the German Aerospace Center (DLR) in Cologne, Germany. Healthy non-smoking adults aged between 24 and 55 years with a body mass index between 19–30 kg/m^2^ and free of any chronic illness and pain, elevated risk of thrombosis or bone fractures less than 1 year prior to the study were recruited. The potential volunteers were further screened for clearly defined medical exclusion criteria such as: chronic hypertension, diabetes, obesity, arthritis, hyperlipidemia, hepatic disease (A, C), or a disorder of calcium or bone metabolism. The subjects were also screened for any eye conditions that could significantly impact visual function. Volunteers that were medically eligible for the study subsequently underwent psychological screening.

Eleven individuals (6 males and 5 females) aged 25.3–50.3 years at the time of study admission participated. They were admitted to the study 14 days prior to the start of the 6° HDBR + CO_2_ intervention which lasted for 30 days. The level of atmospheric CO_2_ enrichment during bed rest (3.8 mmHG ambient partial pressure of carbon dioxide) was selected to match the average CO_2_ concentration onboard the ISS (Law et al., 2014).

All participants strictly adhered to the head down tilt position at all times. Subjects received a controlled diet and were provided with an 8-h sleep opportunity from 10:30 PM until 6:30 AM daily. They participated in multiple other NASA-funded studies during the HDBR + CO_2_ intervention such as ocular, intracranial pressure (ICP) measures and blood gas analysis. Participants were dismissed 14 days following completion of the bed rest phase; they received monetary compensation for their participation. The study procedure was approved by the local ethical commission of the regional medical association (Ärztekammer Nordrhein) as well as the University of Florida and NASA Institutional Review Boards. All subjects provided written informed consent at the German Aerospace Center in Cologne, Germany.

#### HDBR

The methods of our past ambient air HDBR study have been described in previous publications (Koppelmans et al., 2013, 2015, 2017; Cassady et al., 2016; Yuan et al., 2018). The participants were 18 males aged 25.8–39.8 years at the time of admission. These subjects were randomly assigned either to a no-exercise (*n* = 5), an aerobic and resistance exercise (*n* = 5), or a flywheel exercise group (*n* = 8). These groups were pooled for the analyses here.

The demographic information of the HDBR + CO_2_ and HDBR groups as well as the HDBR + CO_2_ subgroups by SANS status is provided in Table 1. All HDBR + CO_2_ and HDBR subjects passed an Air Force Class III equivalent physical examination. None of the participants were exposed to both experimental procedures.

**TABLE 1:** Subject demographic information.

	**HDBR**		**HDBR + CO_2_**			
***N***	**Males = 18**		**Females = 5 Males = 6**			

Age^a^ Mean (SD)	31.13 (4.70)		33.91 (8.03)			
	**Control**	**Exercise**	**SANS**		**NoSANS**	
***N***	**Males = 5**	**Males = 13**	**Female = 3**	**Males = 2**	**Female = 2**	**Males = 4**
Age^a^ Mean (SD)	33.7 (5.4)	30.2 (4.2)	34.5 (3.9)	42.7 (10.8)	35.5 (13.3)	28.3 (11.0)

*HDBR: head down bed rest, SANS: Spaceflight Associated Neuro-ocular Syndrome. ^*a*^Age in years.*

### Testing Timeline

#### HDBR + CO_2_

Behavioral measurements were obtained across six time points: (a) two baseline measurements 13 and 7 days before bed rest; (b) two measurements 7 and 29 days during bed rest; and (c) two measurements 5 and 12 days post bed rest. Functional mobility and balance were not tested during bed rest as they would have required participants to be upright. Instead, these two behaviors were tested on the first day following HDBR + CO_2_ completion; the data were obtained within approximately 3 h of participants first standing up.

#### HDBR

**FIGURE 1:**
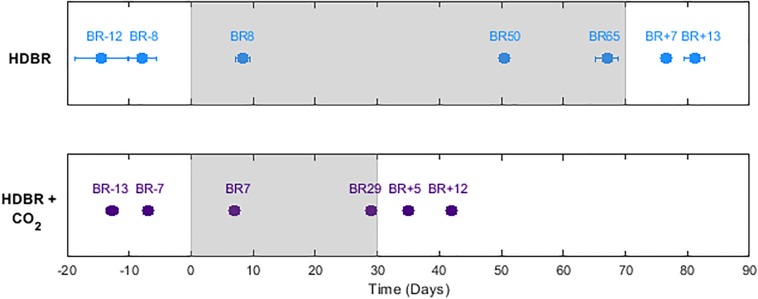
Assessment time line and mean testing days. HDBR + CO_2_ refers to the current data set, while HDBR refers to our previous 70 day bed rest study. BR-days refer to the number of days prior to entering bed rest, BR days are within the bed rest intervention, and BR + days are after exiting bed rest. The postural assessment and functional mobility test were also performed on R + 0.

The assessment timeline of the HDBR subjects has been previously published (Koppelmans et al., 2015). Behavioral measurements were obtained at seven time points: (a) two measurements approximately 12 and 8 days before bed rest; (b) three measurements at approximately 8, 50, and 65 days in bed rest; and (c) the first day of standing up from bed rest for tests requiring upright stance, and days 7 and 13 post bed rest for all tests (see Figure 1 for testing time line and average assessment days for HDBR + CO_2_ and HDBR). Given that the HDBR duration and testing time points differed between the two studies, we compared the slopes of behavioral change over time to assess any additive effects of HDBR + CO_2_ over HDBR.

### Cognitive and Sensorimotor Tests

All tests were identical between the two studies, with the exception of the balance assessments. This will be described further below. A detailed overview of the cognitive and sensorimotor tests can be found in Koppelmans et al. (2013).

#### Neuropsychological Assessments

Standard neuropsychological tests were used to measure several cognitive constructs. The paper-and-pencil version of the digit symbol substitution test (Lezak, 2004) was used to assess processing speed; we determined completion time and accuracy. Thurstone’s 2D card rotation task was administered to measure spatial working memory performance (Ekstrom et al., 1976). The total and correct number of responses made within 3 min were measured as indicators of performance. The Purdue pegboard test was used to evaluate bimanual coordination (Tiffin and Asher, 1948). The time taken to bimanually insert small cylindrical metal pegs into 15 rows of two parallel holes was measured. The testing materials for these three assessments were mounted on a board that could be height-adjusted for each subject so that the subjects could comfortably perform the test in the head down tilt position.

The rod and frame test (RFT) (Kindrat, 2011; Nair et al., 2018) was used to measure reliance on visual vs. other cues (vestibular, proprioceptive) for vertical perception. Each subject’s field dependency and error profile were calculated based on the degrees of deviation between the perceived and true vertical. The rod angle error relative to the actual vertical was measured for each of the trials. Constant error measures the subjects’ average bias in estimation of the true vertical across all trials and is characteristic of each subject (Nyborg, 1974). The frame effect is the main measure of visual dependence, which shows the magnitude of the error in the subjects’ estimate of the vertical caused by the tilted frame regardless of direction of frame or rod tilt, while also taking into account individual subjects’ bias (Nyborg, 1974). Response consistency refers to the within subject variability of scores obtained across the same rod/frame tilt combination. Given that higher values on this measure refer to greater variability, we refer to this variable as response variability from here forward.

Visuospatial processing was assessed using the 3D Cube Mental Rotation Task (Shepard and Metzler, 1988). The response time and accuracy to determine the 3-dimensionally rotated form of the previously seen groups of cubes were indicators of performance. Participants were lying on their side to complete the RFT and mental rotation tasks so that they could view a monitor placed bedside.

#### Single and Dual Tasking

Participants performed a finger tapping motor task under single and dual task conditions (Yuan et al., 2016) while in an MRI scanner; only behavioral results are presented here. Two stimulus boxes were presented on the screen side-by-side. An “x” appeared in one of the two locations randomly with an inter-stimulus interval of 800 ms; subjects were instructed to press the corresponding button with the left or right index finger as quickly as possible. The secondary counting task required subjects to watch a square that was changing colors at a rate of 3 Hz and to count the number of times a target color appeared. Target color appearance was low (1–3%) so that subjects had to remain vigilant. Participants performed the two tasks in isolation and simultaneously. Tap reaction time, tap accuracy and counting accuracy were analyzed as performance measures.

#### Functional Mobility Test

The 6.0 m × 4.0 m functional mobility test (FMT) course consists of several foam obstacles such as hurdles, pylons and bars presenting a series of mobility challenges; the test has been shown to be sensitive to the effects of spaceflight and bed rest (Reschke et al., 2009; Mulavara et al., 2010; Kopplemans et al., 2017). The first half of the FMT course was set up on a hard floor, whereas the second part was set up on a base of medium density foam to increase postural challenges. Participants were instructed to walk through the course as quickly and safely as possible, without touching any of the obstacles. All participants wore the same brand canvas tennis shoes during the mobility tests, and completed the course a total of 10 times. Here, we analyzed the time to complete the first trial as we have found that this first trial is most sensitive to intervention-associated changes in mobility. The completion time of the first (i.e., hard surface) and second (i.e., compliant foam surface) half of the course during the first trial were separately evaluated.

#### Posturography

A posturography test (Black, 2001) was carried out in order to assess the subjects’ ability to control their balance. Details of the test have been previously described in Mulder et al. (2014). The subjects were instructed to maintain a stable upright posture in open stance with their arms folded across their chest for 30 s on a foam pad that rested on a force platform (Leonardo Mechanograph, Novotec Medical GmbH, Pforzheim, Germany). The feet were aligned against the guidelines demarcated on the foam pad in order to maintain consistent foot positioning between and within subjects. Balance control was measured in three conditions: (a) eyes open with the head erect (EO); (b) eyes closed with the head erect (EC); and (c) eyes closed with dynamic head tilts (ECDHT), where the subjects rhythmically made head pitch motions of ±20° synchronized to a 0.33 Hz metronome tone. Each condition was performed three times, where the order of conditions was semi-randomized to ensure no identical conditions repeated back-to-back.

The balance assessment of the HDBR study utilized a computerized dynamic posturography system (Equitest, NeuroCom International, Clackamas, OR, United States) (Reschke et al., 2009). The subjects performed two conditions with their eyes closed, on a sway-referenced base; one with head erect (Sensory Organization Test 5) and another with dynamic head tilts (following a 0.33 Hz-paced audible tone). Each condition consisted of three 20-s trials. Based on the similarity of the balance challenges, only the EC and ECDHT conditions will be statistically analyzed for the HDBR + CO_2_ and HDBR group comparison.

For all three balance control conditions, we selected the median score of the three trials to prevent effects of outliers. The anterior-posterior (AP) peak-to-peak center-of-mass sway angle was used to compute an equilibrium score (EQ).

### Statistical Analyses

#### HDBR + CO_2_

We conducted mixed model linear regression analyses on the HDBR + CO_2_ participants, entering time as a continuous variable to assess the effect of the intervention on performance. We omitted the first time point as a practice session. In addition, we excluded the post-HDBR time points for this initial analysis as the intervention effect was our main interest. We used mixed effect models to control for the correlation between repeat visits of the same individual. Moreover, age and sex were entered as covariates to control for their potential confounding effects.

#### Effects of Group and Time in HDBR + CO_2_ and HDBR Subjects

The intervals between test dates and the overall study duration differed for the HDBR + CO_2_ and HDBR studies. Thus, we entered time as a continuous variable when comparing HDBR + CO_2_ and HDBR effects. Linear mixed model analysis was used to examine group × time differences between HDBR + CO_2_ and HDBR subjects. The subject variable was entered as a random intercept. First, in order to examine the additive effect of CO_2_, we tested the simple effects of time, group, and group by time for each outcome measure. We included data from the second baseline measurement and the next two time points, which were collected within HDBR. We also compared the recovery time course between the two groups. For this analysis, we included data from the last HDBR assessment and the following two post-HDBR time points. We limited investigation of recovery effects to the measurements that exhibited significant HDBR-related group × time interactions. Age at admission, sex and exercise group status were entered as covariates.

For completeness, the abovementioned HDBR + CO_2_ analytic procedures were repeated within the HDBR group. Age and exercise group were entered as covariates to control for their potential confounding effects.

#### Effects of Group and Time in SANS and NoSANS Subjects

The SANS vs. NoSANS subgroup comparison analysis was carried out in a similar fashion to that of the HDBR + CO_2_ vs. HDBR comparison described above. For this analysis, age at admission and sex were entered into the model as covariates.

Restricted maximum likelihood (REML) was used in all linear mixed model analyses because its estimation is less sensitive to small sample bias than traditional maximum likelihood (Van Dongen, 1999). Alpha levels were set at 0.05 for all analyses. SPSS25 was used for all statistical analyses (IBM Corp. Released 2017. IBM SPSS Statistics for Windows, Version 25.0. Armonk, NY: IBM Corp.).

Model residuals were checked for normality using Shapiro–Wilk tests and via visual inspection of Q-Q plots. If distributions deviated from normality, the data were log transformed. In case the log transformation did not improve the normality of the residuals, the analysis was repeated without extreme outlier residual data points exceeding the third quartile. In cases here the models based on the untransformed and transformed data agreed after removing the outliers, we present the results based on the untransformed data for ease of interpretation.

## Results

### HDBR + CO_2_ Intervention Effect

For the HDBR + CO_2_ group, a significant main effect of time was observed for the time to complete the digit symbol substitution task. There was also a significant main effect of time for the completion time, accuracy and percent complete of the total 80-item 2D card rotation task. Additionally, although at a trend level (*p* = 0.05), a main effect of time was observed for the RFT response variability (see Table 2). The time to complete the digit symbol substitution task and the RFT response variability score decreased (RFT within subject responses became less variable) as the HDBR + CO_2_ intervention progressed. The 2D card rotation accuracy and the percentage of completed items increased with time, and the time to complete the task decreased as a function of time spent in the HDBR + CO_2_ environment (Supplementary Figure S1). There was also a significant main effect of time for the digit symbol substitution task accuracy after removing outliers (β = 0.012, *p* = 0.035). A few measures exhibited negatively skewed distributions; the RFT frame effect, single condition counting accuracy and dual condition tapping accuracy were all near ceiling, resulting in skewed distributions. As log transformation did not yield normally distributed residuals for these measures, the results with these tests should be interpreted with caution.

**TABLE 2:** Results from the statistical models evaluating the effect of time for the HDBR + CO_2_ and HDBR groups, and the group × time interaction.

		**HDBR + CO_2_**		**HDBR**		**Both**	
		**Time**		**Time**		**Group × time**	
**Cognitive tests**		**β**	**Sig.**	**β**	**Sig.**	**β**	**Sig.**
DSS	Time (s)	–0.81	**< 0.001**	–0.22	0.06	0.59	**< 0.05**
Pegboard	Time (s)	–0.04	0.52	–0.05	0.06	<0.01	0.88
Cube rotation	Time (s)	–0.01	0.18	–0.01	**< 0.001**	<0.01	0.79
Cube rotation	% correct	–0.15	0.15	–0.02	0.70	0.13	0.22
Card rotation	Time (s)	–0.30	**< 0.01**	–0.06	**< 0.01**	0.14	0.27
Card rotation	% correct	0.11	**< 0.05**	–0.01	0.86	–0.12	0.17
Card rotation	% completed	0.16	**< 0.05**	0.07	0.07	0.16	0.05
RFT	Constant error	0.02	0.68	0.02	0.23	<0.01	0.99
RFT	Frame effect	–0.03	0.49	–0.02	0.21	0.01	0.77
RFT	Variability	–0.03	0.05	0.01	0.28	0.04	**< 0.05**
Button press	Time (s)	<0.01	0.27	<0.01	0.10	<0.01	0.09
Button press	% correct	<0.01	0.90	0.03	0.16	0.04	0.46
Counting	% correct	0.25	0.11	0.13	**< 0.05**	–0.12	0.46
Button press, dual	Time (s)	<0.01	0.32	<0.01	0.18	<0.01	0.14
Button press, dual	% correct	–0.04	0.58	0.04	0.24	0.07	0.33
Counting, dual	% correct	–0.25	0.38	0.12	0.17	0.34	0.19
SWM control	% correct	0.10	0.18	0.15	0.11	0.06	0.76
SWM rotation	% correct	–0.03	0.76	0.08	0.10	0.11	0.31
**Sensorimotor tests**							
FMT total	Time (s)	0.15	**< 0.01**	0.05	**< 0.001**	–0.10	**< 0.05**
FMT 1st half	Time (s)	0.06	**<0.01**	0.02	**< 0.001**	–0.04	**< 0.05**
FMT 2nd half	Time (s)	0.09	**< 0.01**	0.03	**<0.001**	–0.06	**< 0.05**
PGEC	EQ	–0.07	0.22	–0.12	**< 0.001**	–0.05	0.48
PG ECDHT	EQ	–0.07	0.11	–0.28	**< 0.001**	–0.22	0.14

*Significant *p* values are highlighted in bold font. HDBR + CO_2_ analyses were controlled for age and sex; HDBR analyses were controlled for age and exercise and the group × time analyses were controlled for age, sex and exercise status. DSS, digit symbol substitution test; RFT, rod and frame test; SWM, spatial working memory; FMT, functional mobility test; PG, posturography test; EC, eyes closed; ECDHT, eyes closed with dynamic head tilts; EQ, equilibrium score.*

### HDBR + CO_2_ vs. HDBR

**FIGURE 2:**
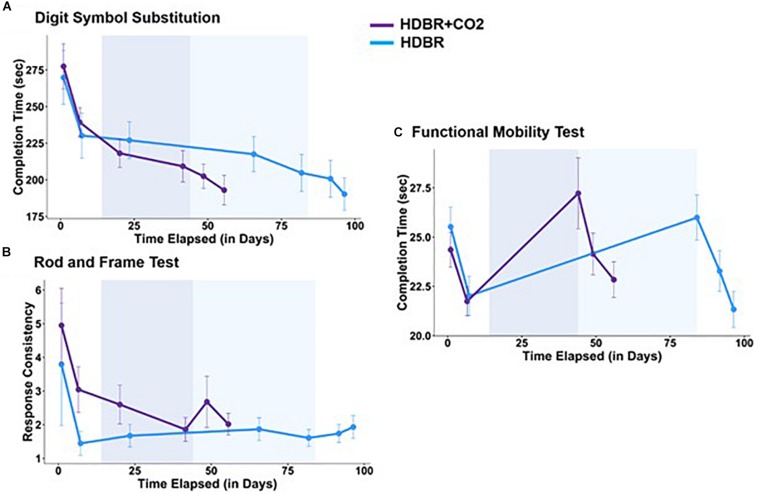
Group by time differential effects during bed rest were observed for **(A)** the digit symbol substitution completion time, **(B)** rod and frame test (RFT) response consistency, and **(C)** the functional mobility test (FMT). No differences were observed in recovery rate. The first two time points on each graph reflect pre bed rest testing. For the digit symbol test and the RFT, the next two time points were collected in bed rest, and the last two after bed rest (the bed rest phase of the HDBR and HDBR + CO_2_ groups are demarcated by shaded boxes of the corresponding color of the groups). For the FMT, the first two time points are pre bed rest and the last three are following bed rest. The error bars indicate SEM.

There was no statistically significant difference in age between the HDBR + CO_2_ and HDBR groups. A significant group by time interaction effect was observed for the digit symbol substitution task completion time (Figure 2A) and RFT response variability (Table 2 and Figure 2B). The degree of performance speed enhancement was greater in the HDBR + CO_2_ group than that of the HDBR group. Similarly, the RFT response variability continued to decrease over time in the HDBR + CO_2_ group while it remained unchanged in the HDBR group. Additionally, significant group by time interaction effects were observed for the total, first and second half FMT completion time (see Figure 2C); the degree of slowing from pre- to post-intervention was greater in the HDBR + CO_2_ group.

None of the measures showed significant group × time interactions post-intervention, supporting that participants recovered their performance following the HDBR + CO_2_ and HDBR interventions at the same rate (Table 3).

**TABLE 3:** Results from the statistical models evaluating the effect of time for the HDBR + CO_2_ and HDBR groups, and the group × time interaction during recovery.

		**HDBR + CO_2_**		**HDBR**		**Both**	
		**Time**		**Time**		**Group × Time**	
		**β**	**Sig.**	**β**	**Sig.**	**β**	**Sig.**
DSS	Time (s)	−1.18	**<0.001**	−0.83	**<0.001**	0.35	0.35
RFT	Variability	0.01	0.79	0.03	0.20	0.01	0.82
FMT total	Time (s)	−0.35	**<0.001**	−0.40	**<0.001**	−0.05	0.65
FMT first half	Time (s)	−0.14	**<0.01**	−0.16	**<0.001**	−0.03	0.56
FMT second	Time (s)	−0.22	**<0.001**	−0.24	**<0.001**	−0.02	0.72

*Only the measurements which showed significant group × time interaction effects as a function of bed rest were tested. Significant *p* values are highlighted in bold font. HDBR + CO_2_ analyses were controlled for age and sex; HDBR analyses were controlled for age and exercise and the group × time analyses were controlled for age, sex and exercise status. DSS, digit symbol substitution test; RFT, rod and frame test; SWM, spatial working memory; FMT, functional mobility test.*

### HDBR + CO_2_ SANS vs. NoSANS

There was no statistically significant age difference between those who developed signs of SANS and those who did not (NoSANS) (see Table 1 and Supplementary Figure S2). Within each sex group, there was also no statistically significant age difference between SANS and NoSANS participants, but it should be noted that stratification into sex and SANS groups greatly reduces the sample size per cell.

**FIGURE 3:**
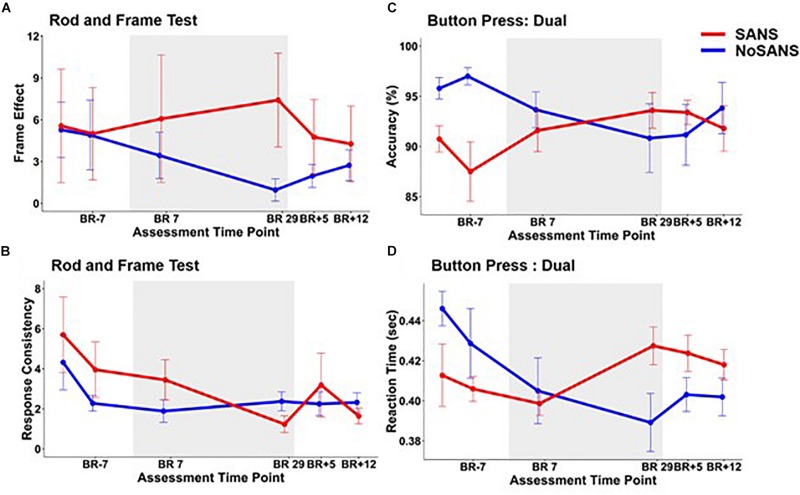
The SANS and NoSANS groups exhibited significantly different changes over time during bed rest on the rod and frame test **(A)** frame effect and **(B)** response consistency measures and also on the dual task button press **(C)** accuracy, and **(D)** reaction time. BR-days refer to the number of days prior to entering bed rest, BR days are within the bed rest intervention, and BR + days are after exiting bed rest. The gray shaded box indicates the time in which subjects were in bed rest. The error bars indicate SEM.

A stratified analysis comparing performance for SANS and NoSANS participants revealed a significant group by time effect on the RFT frame effect (Figure 3A) and response variability (Table 4 and Figure 3B). The results indicated that the SANS group became increasingly visually dependent as the intervention progressed, while the NoSANS subgroup became less so. The RFT response variability for SANS also decreased during HDBR + CO_2_ relative to that of the NoSANS subgroup. Thus the SANS group became more consistently visually dependent.

**TABLE 4:** Results from the statistical models comparing SANS and NoSANS subgroups.

		**Group**		**Time**		**Group × time**	
**Tests**		**β**	**Sig.**	**β**	**Sig.**	**β**	**Sig.**
RFT	Frame effect	−1.68	0.73	0.07	0.26	−0.18	**<0.05**
RFT	Variability	−1.77	0.18	−0.08	**<0.01**	0.09	**<0.01**
Button press	% correct	4.40	0.08	0.08	**<0.01**	−0.15	**<0.01**
Button press, dual	Time (s)	0.03	0.23	<0.01	0.05	−0.01	**<0.01**
Button press, dual	% correct	7.84	**<0.05**	0.15	0.08	−0.32	**<0.01**
PGEO	EQ	−0.12	0.92	0.01	0.64	−0.08	**<0.05**

*Only the tests resulting in significant group × time interaction effects are listed. Significant *p* values are highlighted in bold font. The analyses are controlled for age and sex. RFT, rod and frame test; PG, posturography test; EO, eyes open; EQ, equilibrium score.*

There was also a significant subgroup by time interaction for finger tapping accuracy under both single and dual task conditions and for finger tapping reaction time under the dual task condition. In both single and dual task conditions the accuracy of performance for the SANS subgroup increased, while the NoSANS subgroup decreased during the intervention (Figure 3C). The SANS subgroup dual task reaction time slowed down as the intervention progressed as opposed to the facilitation observed in the NoSANS group (Figure 3D).

**FIGURE 4:**
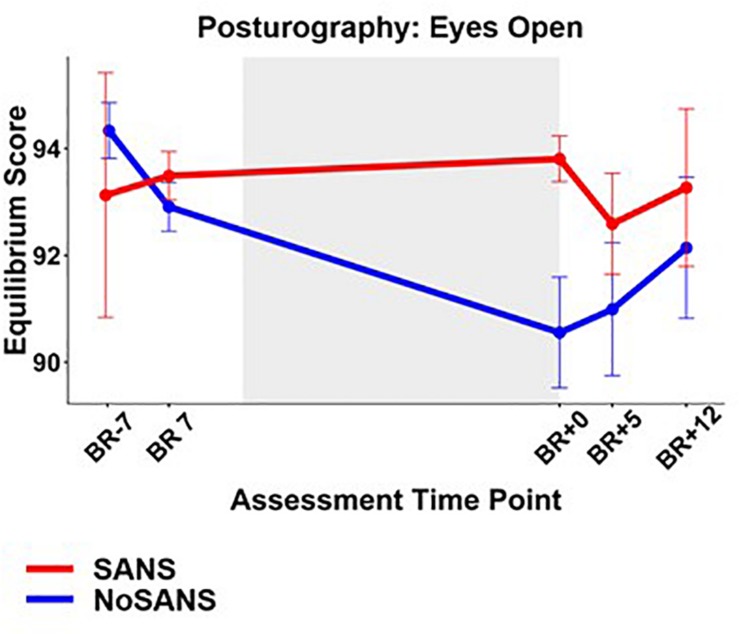
The SANS and NoSANS groups exhibited significantly different changes from pre- to post HDBR + CO_2_ on the balance performance of the eyes open condition of the posturography. BR-days refer to the number of days prior to entering bed rest, BR days are within the bed rest intervention, and BR + days are after exiting bed rest. The gray shaded box indicates the time in which subjects were in bed rest. The error bars indicate SEM.

A significant group by time interaction was observed in the eyes open, head erect (EO) posturography test condition. In comparison to the NoSANS group, the SANS group showed less decrements in postural control in this EO condition following the HDBR + CO_2_ intervention (Figure 4).

## Discussion

Here we investigated the combined effects of HDBR and increased CO_2_ concentration on a range of cognitive and sensorimotor assessments. We found a greater facilitation of processing speed and larger decrements in functional mobility relative to our previous HDBR study. The HDBR and HDBR + CO_2_ groups did not differ in digit symbol and functional mobility performance change over time post bed rest, suggesting an HDBR + CO_2_ specific facilitation of processing speed and decline of functional mobility, with recovery occurring at the same rate once subjects were out of the HDBR + CO_2_ and HDBR environments.

Although the HDBR + CO_2_ subjects showed improving performance in the 2D card rotation task, the degree of change was not greater than that of the HDBR group which showed similar patterns of change. This performance change seen in the HDBR + CO_2_ group would most likely reflect practice effects. Our finding of faster processing speed in the HDBR + CO_2_ subjects in comparison to the HDBR subjects is in line with previous reports of enhanced cognition under short duration (i.e., 26 h) 12° HDBR combined with 0.5% CO_2_ (Basner et al., 2018). Astronauts have also exhibited processing speed changes inflight, which fluctuate with reported arousal levels (Kelly et al., 2005). Although the exact CO_2_ level and response of the astronauts in that report are difficult to determine, increased vigilance and arousal as a result of CO_2_ exposure (Guyenet et al., 2010) in the current protocol may have contributed to the facilitation of processing speed. It is also possible that the cerebral vasodilation that occurs with elevated CO_2_ (Sliwka et al., 1998), which selectively favors frontal lobe perfusion (Bhogal et al., 2015), could have resulted in the enhanced processing speed here. This is particularly the case given frontal involvement in processing speed and efficiency (Rypma et al., 2006). One caveat however is that participants in the current study did not demonstrate a significant change in end-tidal PCO_2_ from beginning to end of the intervention (Laurie et al., 2019). This is difficult to interpret, however, given that these measures were not collected throughout the HDBR + CO_2_ campaign. Thus the lack of change might reflect adaptation over time.

The RFT, which measures an individual’s vertical perception (Witkin et al., 1954), also yields measures of RFT response variability. While the HDBR group maintained low response variability throughout the study, the HDBR + CO_2_ group demonstrated progressive decreases in response variability. Typically, more variable responses on this task indicate subject disorientation in the RFT (Nyborg, 1974). It is noteworthy that despite the baseline difference where the HDBR + CO_2_ group showed less consistent responses, this variability decreased as a function of time spent in the HDBR + CO_2_ condition.

The HDBR + CO_2_ intervention resulted in significant slowing to traverse the entire, first, and second half of the FMT course. This is in line with a previous long term bed rest intervention (Koppelmans et al., 2015) and spaceflight effects (Mulavara et al., 2010), which also led to declines in functional mobility. While reduced loading and usage of the lower limbs and sensory reweighting are the proposed major factors driving such findings (Lee et al., 2019), it is interesting that a greater degree of decrement was observed in the HDBR + CO_2_ group in comparison to HDBR, despite the relatively shorter intervention duration. Although the potentially protective effects of exercise in some of the HDBR group may have impacted these findings, this is unlikely to be the main driving force as we covaried for the exercise status in our analyses. Unlike the facilitation of psychomotor speed, the addition of elevated CO_2_ to HDBR led to a decrements in whole body functional mobility.

### Differential Changes for SANS vs. NoSANS

Previous bed rest studies have not observed signs of SANS; the fact that five individuals did here allowed us to compare SANS and NoSANS subgroups (Laurie et al., 2019). This is important, as SANS has recently been shown to affect approximately 1/3 of crewmembers participating in missions of 6 months or longer (Lee et al., 2016). These two subgroups differed on several of our measures. Performance on the RFT relies on multisensory integration processes (Bronstein, 1999; Isableu et al., 2008; Lopez et al., 2008). Interestingly, posture control involves similar processes (Massion, 1994) where alteration in one sensory input results in multisensory reweighting (Ernst and Bulthoff, 2004). Moreover, field-dependent individuals who tend to base their perception of verticality on external cues are less stable and more dependent on visual cues for balance than individuals who are field independent. In line with these characteristics, we found that SANS individuals increased their reliance on visual cues during the HDBR + CO_2_ intervention. The NoSANS subgroup, in contrast, became more field-independent. In addition, individuals in the SANS subgroup decreased their response variability during the intervention. The SANS subgroup also showed higher levels of balance control in the eyes open condition. This profile of increased visual dependency with SANS is worth noting; as visual dependency has been associated with poorer navigation (Willey and Jackson, 2014) this may impact the re-adaptive process of astronaut. While it may seem counterintuitive to have ocular declines associated with increased reliance on vision, the signs of SANS here were quite mild in comparison to what is seen with spaceflight. The participants here exhibited ocular disc edema, whereas astronauts with SANS also have visual acuity changes, globe flattening, and other ocular structural changes. Perhaps the increased reliance on vision is a compensatory response to the early stages of SANS development.

The SANS subgroup showed increased finger tapping accuracy in both the single and dual task conditions while accuracy declined for the NoSANS group. Slowing in the SANS group may reflect an implicit strategy to maintain high levels of accuracy.

### Limitations

Some of the opposing changes we observed in the SANS and NoSANS subgroups may have hindered our ability to detect overall group effects in response to HDBR + CO_2_. Moreover, the sample size of the HDBR + CO_2_ group is quite small (*n* = 11); replication should be performed before the results are broadly generalized. In addition, having a control group with the same testing schedule would help to better quantify changes.

This is the first time that symptoms of SANS have been reported in a spaceflight analog environment. While our findings are compelling and suggest implications for mission relevant behaviors, further investigation is warranted to determine whether these symptoms are comparable to the syndrome as manifested in astronauts.

## Conclusion

The addition of elevated ambient carbon dioxide at 0.5% concentration may be protective against long-term HDBR induced processing speed deficits. However, the combination of HDBR + CO_2_ may exacerbate the HDBR-associated degradation of multisensory integration. Interestingly, exposure to HDBR + CO_2_ elicited differential physiological responses across individuals, resulting in a subgroup that exhibited signs of SANS. Moreover, we observed differential performance profiles between SANS and NoSANS subgroups, where SANS individuals showed an increased reliance on visual cues and a shift in speed-accuracy trade off. Although these results were obtained with a small sample, they suggest that SANS may negatively impact mission relevant performance. Furthermore, as this study shares implications for home-bound patients with air quality or respiratory concerns, the information and benefits resulting from this project may be far-reaching.

## Data Availability Statement

The datasets generated for this study are available on request to the corresponding author.

## Ethics Statement

The studies involving human participants were reviewed and approved by the Ärztekammer Nordrhein as well as the University of Florida and NASA Institutional Review Boards. The patients/participants provided their written informed consent to participate in this study.

## Author Contributions

RS, AM, and JB designed the study and secured the funding. JL, YD, and IK contributed to the data collection and analyses. JL drafted the manuscript and all authors participated in editing.

## Conflict of Interest

The authors declare that the research was conducted in the absence of any commercial or financial relationships that could be construed as a potential conflict of interest. The reviewer RG declared a past co-authorship with the authors YD, IK, AM, JB, and RS to the handling Editor.
