# Supplementary figures and images for: Head Down Tilt Bed Rest Plus Elevated CO2 as a Spaceflight Analog: Effects on Cognitive and Sensorimotor Performance
Source: Front Hum Neurosci. 2019 Oct 17;13:355. doi: 10.3389/fnhum.2019.00355 (PMC6811492; doi:10.3389/fnhum.2019.00355)

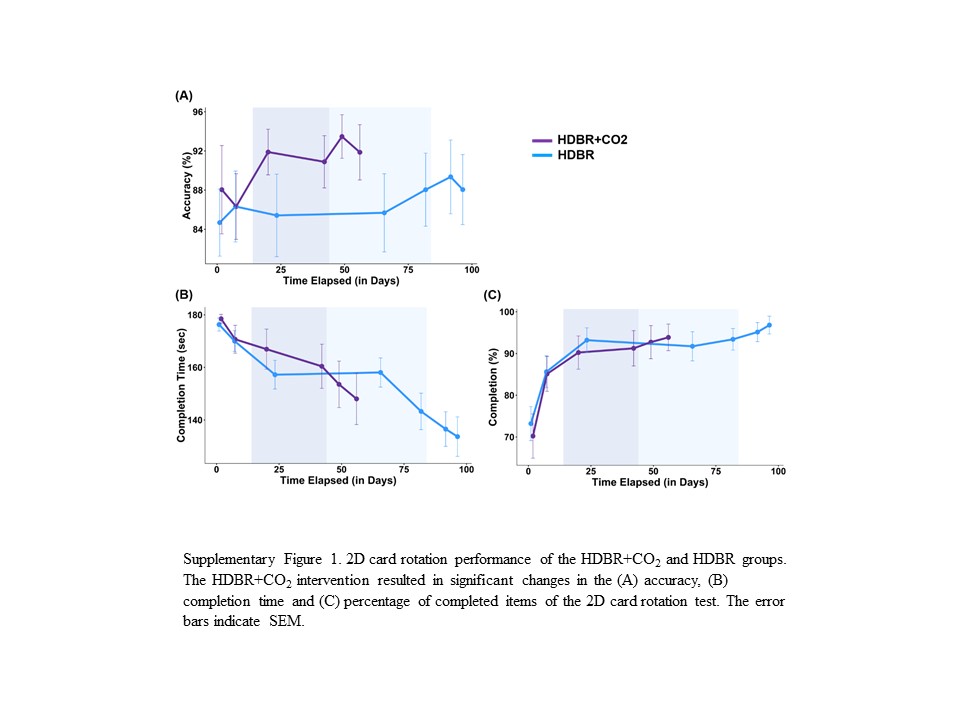

Supplement: Supplementary file 1 [file Image_1.JPEG]

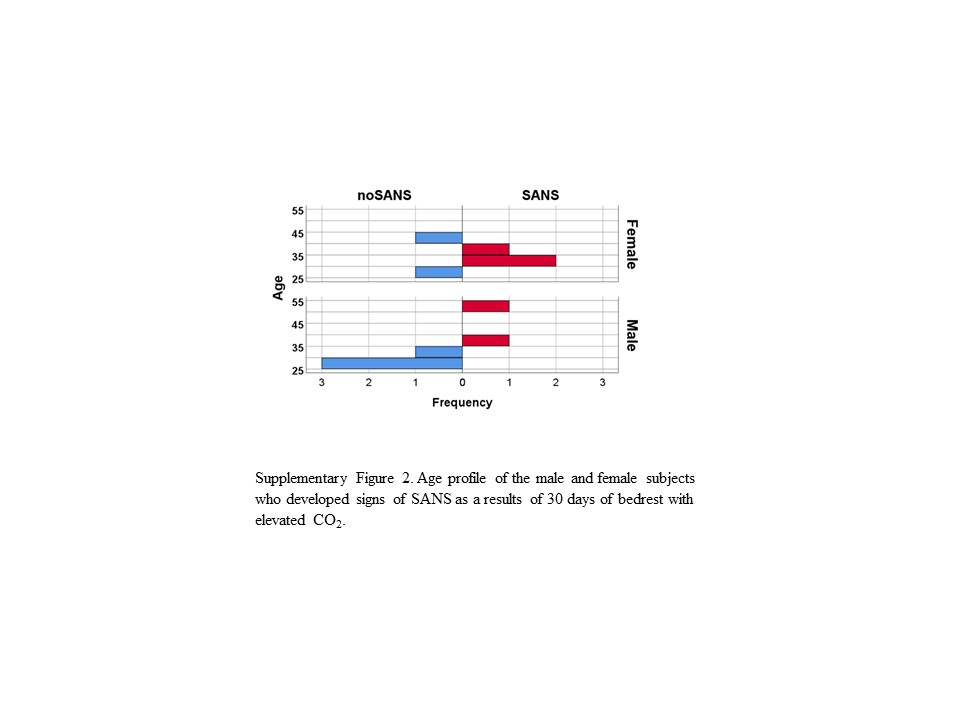

Supplement: Supplementary file 2 [file Image_2.JPEG]
